# Effects of tapering on performance in endurance athletes: A systematic review and meta-analysis

**DOI:** 10.1371/journal.pone.0282838

**Published:** 2023-05-10

**Authors:** Zhiqiang Wang, Yong “Tai” Wang, Weifeng Gao, Yaping Zhong

**Affiliations:** 1 Sports Big-data Research Center, Wuhan Sports University, Wuhan, HuBei, China; 2 Hubei Sports and Health Research Center, Wuhan, HuBei, China; 3 Rochester Institute of Technology, College of Health Sciences and Technology, New York, Rochester, United States of America; University of Bourgogne France Comté, FRANCE

## Abstract

**Objective:**

To assess the responses to taper in endurance athletes using meta-analysis.

**Methods:**

Systematic searches were conducted in China National Knowledge Infrastructure, PubMed, Web of Science, SPORTDiscus, and EMBASE databases. Standardized mean difference (SMD) and 95% confidence interval (CI) of outcome measures were calculated as effect sizes.

**Results:**

14 studies were included in this meta-analysis. Significant improvements were found between pre- and post-tapering in time–trial (TT) performance (SMD = −0.45; *P* < 0.05) and time to exhaustion (TTE) performance (SMD = 1.28; *P* < 0.05). However, There were no improvements in maximal oxygen consumption ($\dot{V}O_{2\max}$) and economy of movement (EM) (*P* > 0.05) between pre- and post-tapering. Further subgroup analysis showed that tapering combined with pre-taper overload training had a more significant effect on TT performance than conventional tapering (*P* < 0.05). A tapering strategy that reduced training volume by 41–60%, maintained training intensity and frequency, lasted ≤7 days, 8–14 days, or 15–21 days, used a progressive or step taper could significantly improve TT performance (*P* < 0.05).

**Conclusions:**

The tapering applied in conjunction with pre-taper overload training seems to be more conducive to maximize performance gains. Current evidence suggests that a ≤21-day taper, in which training volume is progressively reduced by 41–60% without changing training intensity or frequency, is an effective tapering strategy.

## Introduction

The taper is a pre-competition training strategy that reduces training load in a progressive, non-linear manner to induce optimal athletic performance at the right moment of the season [1]. Based on positive (adaptations) and negative (fatigue) effects of training load on the human body [2], the taper aims to reduce negative impact and increase positive training-induced adaptations [3,4], by reducing training load to balance the antagonistic effects of accumulated fatigue and training-induced adaptations [5]. Since 1985, when Costill et al. [6] first experimentally evaluated the impact of pre-competition training load reduction in swimmers, investigators have conducted numerous experimental studies on swimmers [7–9], cyclists [10,11], and runners [12,13] to verify the effect of pre-competition taper on the athletic performance based on physiological and psychological factors affecting such performance, and to explore tapering strategies for different sports.

The key to the pre-competition taper as a typical strategy for improving the performance of endurance athletes is to integrate a scientific arrangement of training variables such as training intensity, volume, frequency, duration of taper, and type of taper [1,14]. Most of the studies confirm that their designed pre-competition tapering strategies could improve sports performance [15–18]. However, researchers disagree on which variables and how much to change. Jafer et al. [19] found that a 50% reduction in training volume significantly improved long-distance runners’ performance. However, some studies have concluded that training volume should be reduced even more, by at least 60%, to significantly improve the athletic performance of endurance athletes in competitions [20]. In addition, a small number of studies have modified pre-competition training load by reducing intensity [10,21]. These studies vary widely on taper duration, ranging from 7 to 28 days. Most studies have used a duration of 8–14-day taper [22], but some have used that a shorter (≤ 7 days) [23] or longer (> 28 days) [24] duration. However, all these durations of taper can yield positive training effects for athletic competitions. In addition, the types of tapering including step and progressive tapers have been proven effective [25], however, the progressive taper appears to be more successful [26]. In summary, pre-competition tapering methods in the aforementioned studies differ significantly, and it is still unclear about which tapering are better at improving the performance of endurance athletes.

Previous meta-analyses (249 swimmers, 80 road cyclists, and 110 track runners) confirmed the benefits of pre-competition tapering on improving sports performance and suggested that a duration of 14-day, with no change in training intensity nor frequency, but with training volume exponentially reduced by 41–60%, appears to be the most effective pre-competition tapering strategy [27]. Despite this meta-analysis evaluating the effects of taper component changes on performance in competitive athletes, the limitation is that this study focused not only on endurance athletes but also sprinters. We did not perform a comparative analysis of the reported type of sports event, and therefore the authors could not provide more specific recommendations on endurance sports. Given the differences sport-to-sport and athlete-to-athlete, these findings might not accurately reflect the characteristics of tapering for endurance sports. Although the application effects of tapering in endurance events have received much attention in the last decade [13,22,28–31], it is very difficult for athletes, coaches, and researchers to find tapering strategies to increase performance in endurance athletes. The previous systematic reviews have helped to shed light on the effects of tapering strategies on competitive performance [30,32], and on nutrition, hydration, and recovery strategies during pre-competition taper [30]. However, these reviews did not address typical indicators used to evaluate specific sports and aerobic capacities, such as maximal oxygen consumption ($\dot{V}O_{2\max}$), time to exhaustion (TTE), and economy of movement (EM), which determine endurance performance [33–38]. These limitations have a significant impact on how tapering measures are interpreted to utilize effective evidence-based practices for endurance athletes. Therefore, it is very difficult for athletes, coaches, and researchers to identify appropriate tapering strategies to enhance performance in endurance athletes. It seems necessary to perform a systematic review and meta-analysis of the literature on the effect of tapering on endurance performance.

A TT test is defined as an endurance performance test with a known endpoint. In this type of test, subjects are required to complete a set distance in as fast a time as possible [37]. TT is the measurement of the time taken to complete a given distance (e.g., a race or time trial) [36]. It can effectively simulate the physiological responses of athletes during competition, is often used to evaluate the specific performance of athletes, and is closely related to endurance performance [36,39]. TTE, $\dot{V}O2\max$, and EM are typical indicators that objectively and directly reflect specific exercise and aerobic capabilities [34,37,39,40]. The TTE test requires subjects to perform submaximal exercise intensity to exhaustion until they no longer maintain the required work rate (e.g., speed or power output) [33,37]. $\dot{V}O2\max$ is defined as the maximal oxygen consumption rate measured during incremental exercise [41]. EM is defined as the oxygen consumption during exercise to generate a given running speed or cycling power output at a given submaximal exercise intensity [4,38].

The purpose of this systematic review and meta-analysis was to assess the effects of tapering on TT, TTE, $\dot{V}O_{2\max}$, and EM, all of which are known to be determinants of endurance sport performance [33–38]. Furthermore, a subgroup meta-analysis was conducted to summarize the characteristics of training intensity, training volume, frequency, duration, and type of pre-competition taper to determine an optimal pre-competition tapering strategy and provide more feasible guidance for training before endurance conoetitions.

## Methods

This systematic review was conducted following the guidelines of the Preferred Reporting Items for Systematic Review and Meta-Analysis (PRISMA) [42].

### Literature search

Two independent reviewers (WZQ and GWF) performed the search in the following databases: China National Knowledge Infrastructure (CNKI), PubMed, Web of Science, SPORTDiscuss, and EMBASE. An electronic search was conducted that included all publication years (up to and including September 18, 2022). Reviewers used the following Boolean search phrases in all of the above-mentioned databases: (taper*) AND (endurance training OR endurance exercise* OR swim* OR cycli* OR runn* OR rowi* OR race walk* OR ski* OR skat* OR sport* OR exercise*) AND (performance* OR competition* OR training). In addition to the primary search, we also performed a secondary search that included checking the reference lists of all included studies. Search terms were obtained from reviews of previous studies and from common synonyms used in discussions of tapering and endurance events.

### Eligibility criteria

We included studies that fulfilled the following criteria: 1) Participants: subjects were endurance athletes ($\dot{V}O_{2\max}$ > 55 ml/kg/min) [43]. If the included studies did not provide $\dot{V}O_{2\max}$ values, training status was determined by the classification used in each study, and the participants were at least well-trained athletes [44,45]; 2) Interventions: the experimental design provided detailed pre-competition tapering data, including training intensity, volume, frequency, type, and duration. A taper is a short-term (≥6 days [46,47]) reduction in training load (e.g., the alteration of training volume, intensity, and frequency); 3) Study design: randomized controlled trial (RCT) or controlled trial [48,49]; 4) Outcome indicators: TT tests that required participants to complete a set distance, TTE, $\dot{V}O_{2\max}$, and EM. Measurements of TT and TTE refer to the total time desired to achieve the whole goal quantity, including direct measurements such as performance during either a constant distance (at least middle distance) or a constant intensity test. We chose TT as the primary-outcome index for examining athletic performance, and the endurance performance–related indices TTE, $\dot{V}O2\max$, and EM as secondary-outcome indices, to assess the effects of pre-competition taper on sports performance in endurance events; and 5) Quality assessment: at least moderate study quality on the Physiotherapy Evidence Database (PEDro) scale or better (> 4 points) [50–52].

Exclusion criteria were as follows: 1) experimental data could not be extracted and remained unavailable after we contacted the authors; 2) the full text of the study was unavailable; 3) taper duration was less than 6 days; 4) published in a non-English language; or 5) TT not based on distance. Time-based TT tests (e.g., a 60-minute time trial or a 5-minute TT), in which an athlete attempts to travel the furthest distance or to maintain the highest average power or velocity within a set duration, are common. However, relative to time-based TT tests, TT tests that require participants to complete a set distance are the most common type of TT test, which most closely represent a true race environment. They may be the most appropriate for endurance performance assessment [53].

### Study selection and methodological quality assessment

The lead author removed duplicates of articles identified across numerous search databases and screened the titles and abstracts of the search results. If a definitive decision could not be made, studies were taken forward for a full study review. The eligibility of the full-text articles was determined according to the inclusion and exclusion criteria by two authors independently. A discussion between the two authors resolved any disagreements regarding eligibility. If necessary, a third researcher was consulted to reach a consensus.

We used the PEDro scale to evaluate study quality. This scale has a total of 11 scoring points ranged from 0 to 10. Scoring is as follows: 9–10 points indicate high quality, 6–8 points indicate slightly high quality, 4–5 points indicate moderate quality, and < 4 points indicate low quality. Only studies with better than moderate quality were included [50,51].

### Data extraction

The two reviewers (WZQ and GWF) independently extracted information from each study, including the characteristics of participants, tapering strategies, and outcome data (Table 1):

1. Participants: age, sex, sample size, training status, and TT distance.
2. Interventions: training intensity, volume, frequency, duration, type of tapering. In accordance with the meta-analysis conducted by Bosquet et al. [27], we coded training intensity (decreased or unchanged), volume (≤ 20%, 21–40%, 41–60%, or ≥ 60%), frequency (decreased or unchanged), duration of taper (≤ 7 days, 8–14 days, 15–21 days, or ≥ 22 days), and type of tapering (progressive or step).
3. Outcome data: pre- and post-tapering mean ± standard deviation (SD) of TT, TTE, and $\dot{V}O_{2\max}$. If only standard error (SE) was reported in the study, we used the formula SD = SE × √*N* [54].

**Table 1:** Characteristics and outcomes of included studies.

Study	Age	M/F	Volume (%)	Intensity (%)	Frequency (%)	Duration (days)	Type	Outcomes	PEDro scores
**Houmard *et al*. [15]**	32.0±2.6	10/0	−70	U	−17	21	S	TT, TTE, EM $\dot{V}O_{2\max}$	5
**McConnell *et al*. [55]**	31.6±1.4	10/0	−66	−20	−50	28	S	TT, TTE EM $\dot{V}O_{2\max}$	5
**Houmard *et al*.** **[56]**	28.3±9.51	12/4	−85	U	U	7	P	TT, TTE, $\dot{V}O_{2\max}$	6
**Child *et al*. [57]**	30.5±1.61	14/0	−85	U	−14	6	P	TT	6
**Mujika *et al*. [46]**	19.9±1.8	8/0	−75	U	U	6	P	TT	6
			−50	U	U	6	P		
**Mujika *et al*. [58]**	19.4±3.2	9/0	−69	U	U	6	P	TT	6
			−65	U	−33	6	P		
**Neary *et al*. [10]**	22.6±4.7	11/0	−30	U	U	7	S	TT	6
			−50	U	U	7	S		
			−80	U	U	7	S		
**Neary *et al*. [16]**	25±6	22/0	−48	U	U	7	P	TT, $\dot{V}O_{2\max}$	6
			U	−20	U	7	P		
**Neary *et al*. [17]**	22.6±4.7	11/0	−30	U	U	7	S	TT	6
			−50	U	U	7	S		
			−80	U	U	7	S		
**Luden *et al*. [14]**	20±1	7/0	−50	U	U	21	S	TT, $\dot{V}O_{2\max}$, EM	5
**Ishak *et al*. [59]**	16.9±0.8	27/0	−43	U	U	14	P	TT, $\dot{V}O_{2\max}$	6
			−46.5	U	U	14	P		
**Skovgaard *et al*. [13]**	29.2±4.5	8/3	−49	U	U	18	S	TT, TTE, EM	5
**Spilsbury *et al*. [30]**	21.7±3.0	10/0	-30	U	-10	7	P	TT	6
**Spilsbury *et al*. [60]**	21.4±4.2	8/0	-32	U	-18	7	P	TT	5
			-56	U	-18	7	P	TT	5

U = unchanged; S = step; P = progressive; TT = time trial; TTE = time to exhaustion; $\dot{V}O_{2\max}$ = maximum rate of oxygen consumption measured during incremental exercise; EM = economy of movement.

### Statistical analysis

We used Review Manager (RevMan) version 5.4 (Cochrane Collaboration, Oxford, UK) for heterogeneity assessment, sensitivity analysis, synthesis of results, subgroup analysis, and forest map creation. Because this study included both RCTs and controlled trials, and also included different types of endurance events, we used the inverse-variance method for meta-analysis based on type of experimental design, in accordance with the *Cochrane Handbook for Systematic Reviews of Interventions* [61]. Standardized mean difference (SMD) and 95% confidence interval (CI) of TT, TTE, $\dot{V}O_{2\max}$, and EM outcomes were calculated as effect sizes. Heterogeneity was quantitatively evaluated by *I*^2^ values: 25% (low), 50% (moderate), and 75% (high) [62]. We used the *Q* statistic to test heterogeneity. The *P*-value for *χ*^2^ was < 0.1, indicating whether a study showed statistically significant levels of heterogeneity [63].

Two investigators (WZQ and GWF) independently assessed the studies’ risk of bias by interpreting the funnel in the Begg and Egger tests using STATA software version 16.0 (Stata Corp., College Station, TX, USA).

## Results

### Characteristics of included studies

**Fig 1:**
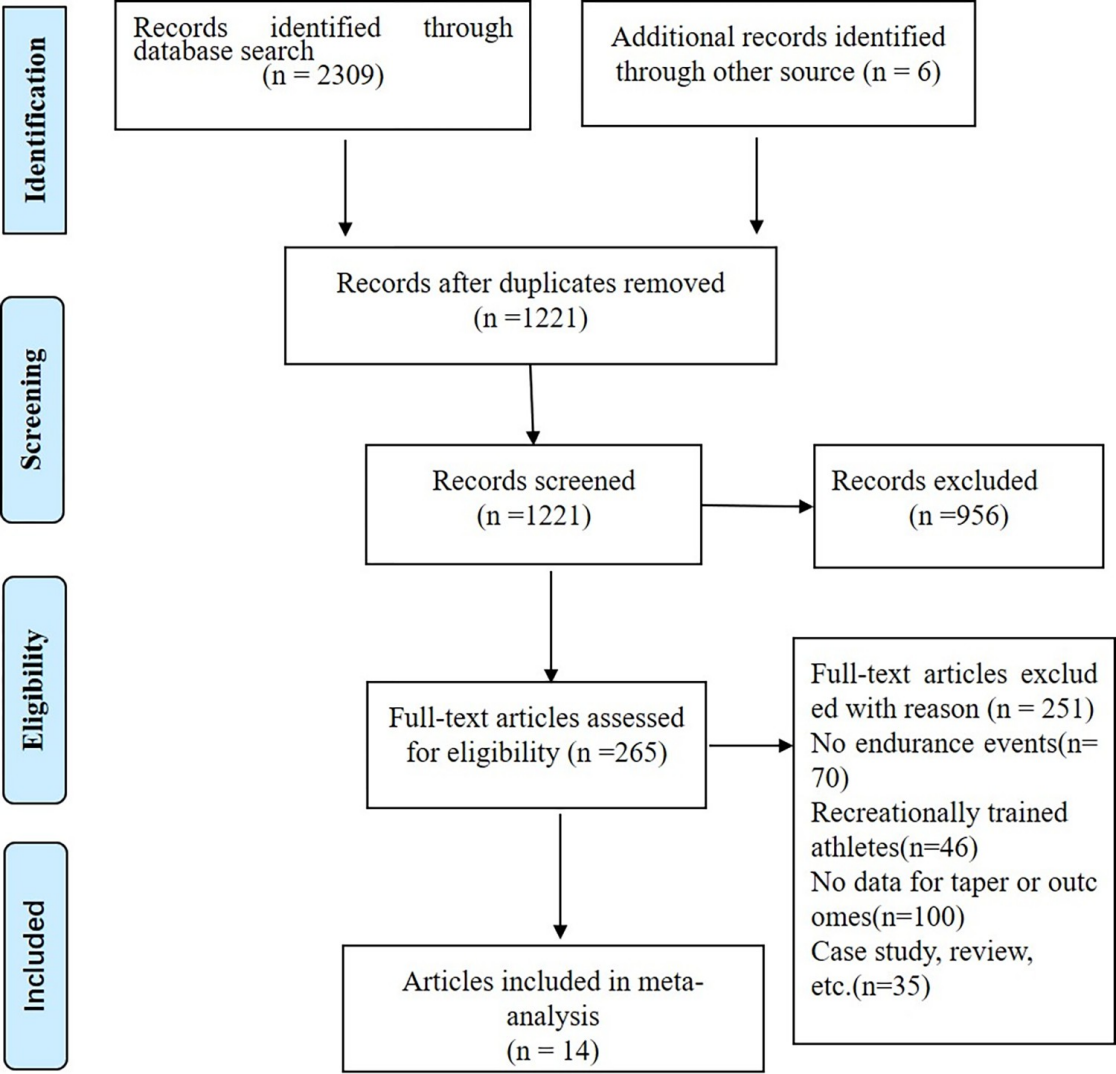
Flow chart of study selection.

A total of 2309 studies were identified with an additional 6 studies included from other sources. After excluding duplicates and screening the title, abstract, or full text, we ultimately included 14 studies in this meta-analysis (Fig 1). These 14 studies included 174 athletes, aged 17–32 years. Their sports were mainly middle-distance, long-distance, and ultra distance running, as well as cycling. We extracted TT data from all 14 studies, 4 studies provided TTE data, 8 studies provided $\dot{V}O_{2\max}$ data, and 6 studies provided EM data (Table 1). In the included study, TT was measured at different distances, ranging from 800 m to 40 km. Performance was assessed on the track, treadmill, and cyclcle ergometer in the selected studies.

Table 1 shows the details of different tapering strategies included in this meta-analysis. Multiple information from the same eligible study needs to be collated. Twenty-one data sets maintained training intensities [10(3), 13, 14, 15, 16, 17(3), 30, 46(2), 58(2), 59(2), 60(2), 61, 62], while two decreased it [16,55]. The range of training volume reduction was ≤ 85%. One data set reduced volume by ≤ 20% [16], four data sets by 21–40% [10,17,30,60], nine data sets by 41–60% [10, 13, 14, 16, 17, 46, 58(2), 60], and nine data sets by ≥ 60% [10, 15, 17, 46, 59(2), 63, 61, 62]. Regarding the reduction of volume, different training methods were usually applied, such as a progressive reduction in training distance, a step reduction in both training duration and frequency, and a step reduction in training hours during tapering days. Training frequency was maintained in sixteen data sets [10(3), 13, 14, 16(2), 17(3), 46(2), 58(2), 59, 61] and decreased in seven. The shortest duration of taper was 6 days, the longest 28 days; seventeen data sets used ≤ 7 days [10(3), 16(2), 17(3), 30, 46(2), 59(2), 60(2), 61, 62], two data sets used 8–14 days [58(2)], three data sets used 15–21 days [15, 58(2)], and one data set used ≥ 22 days [64]. Ten data sets used a step taper [10(3), 13, 14, 15, 17(3), 63], and thirteen data sets used a progressive taper [16(2), 30, 46(2), 58(2), 59(2), 60(2), 61, 62].

### Study quality assessment

Table 1 shows the results of the quality assessment. Study quality ranged from 5 to 6 points. Five studies were classified as being of moderate quality, while nine were considered to have slightly high quality.

### Synthesis of results

#### Time trial performance

**Fig 2:**
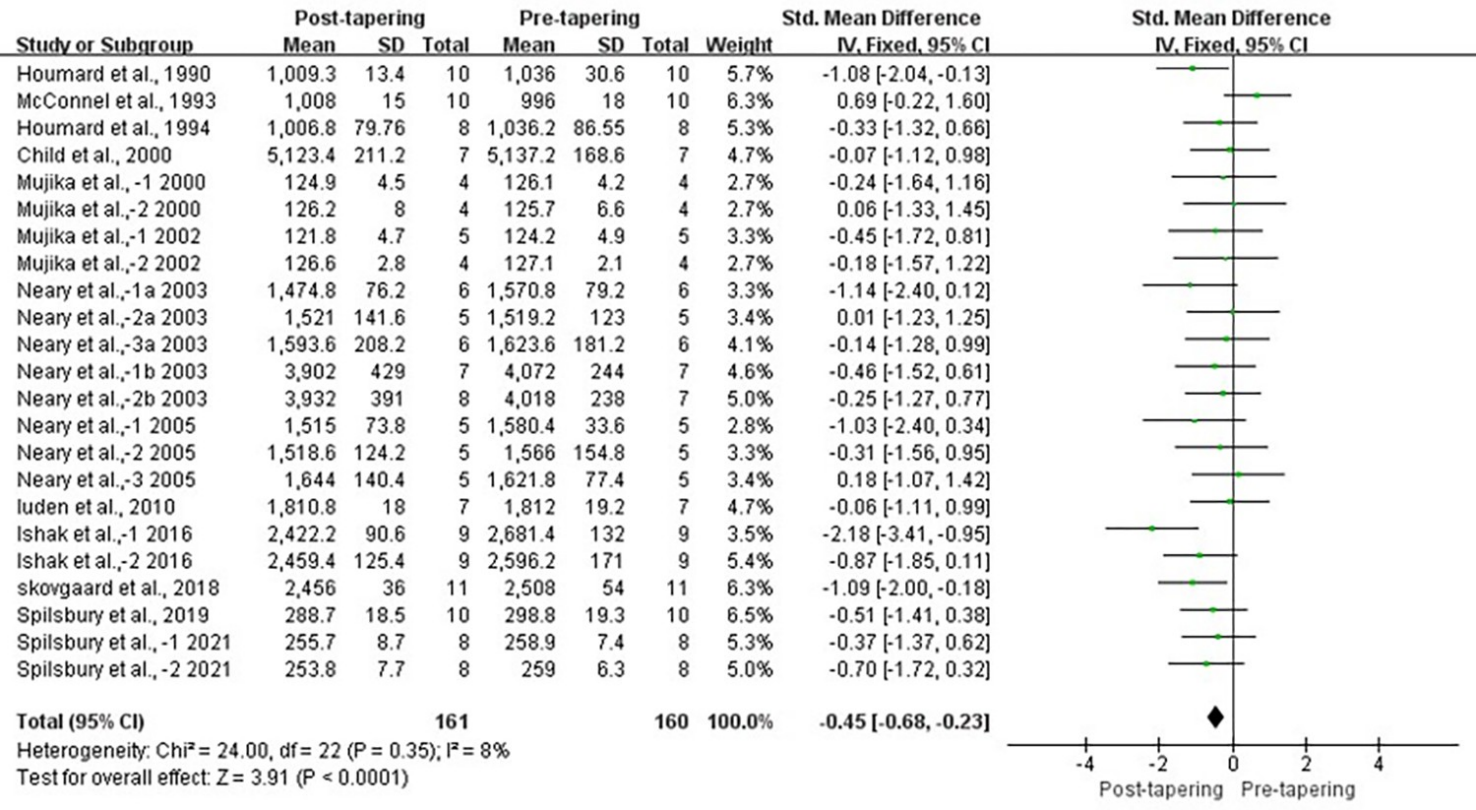
Forest plot of effect size between pre- and post-tapering for TT.

**Fig 3:**
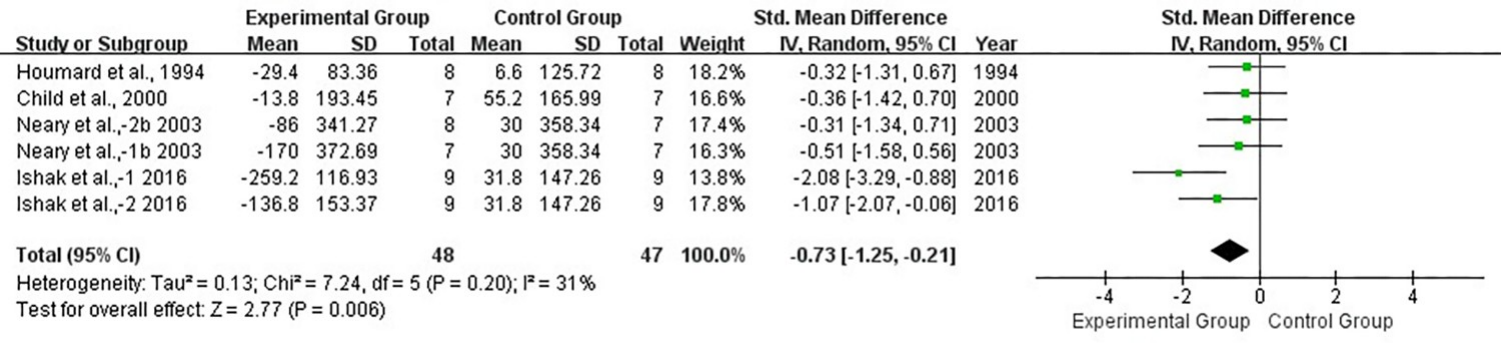
Forest plot of effect size between experimental and control athletes for TT.

Fourteen studies reported data for TT. Levels of heterogeneity were low among these studies (*I*^2^ = 8%, *P* > 0.1). Results for overall effects on TT performance showed a significant improvement between pre- and post-tapering (SMD, −0.45; 95% CI, −0.68 to −0.23; *P* < 0.05; Fig 2). Significant improvement was also observed in experimental groups compared with control groups (SMD, −0.73; 95% CI, −1.25 to −0.21; *P* < 0.05; Fig 3), with a low level of heterogeneity (*I*^2^ = 31%, *P* > 0.1). Begg and Egger tests results showed no significant publication bias in the experimental group for TT (*P* > 0.1).

**Fig 4:**
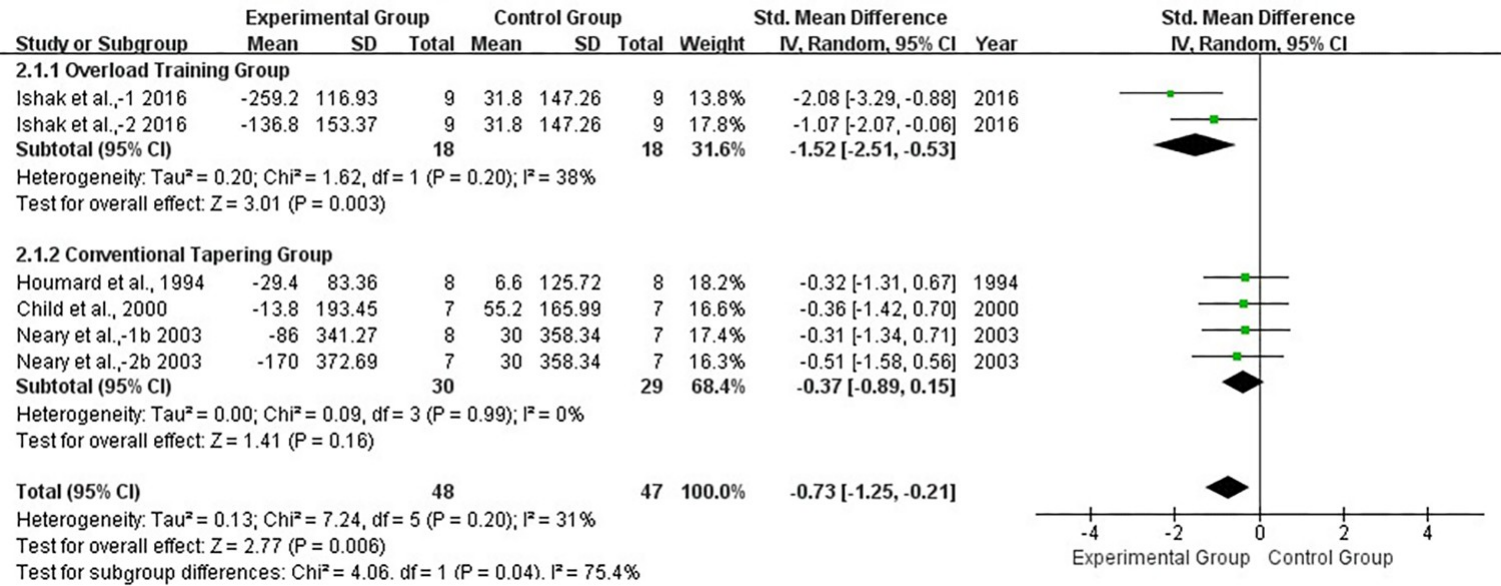
Forest plot of effect size between overload training group and conventional tapering group athletes for TT.

Subgroup analysis with or without overload training before taper showed significantly lower heterogeneity in both subgroups, with *I*^2^ = 0% (*P* > 0.1) in both the overload training group and conventional taper group. This indicated that pre-taper overload training might have been the primary source of heterogeneity. We observed a significant difference between groups when we compared the overload training group with the conventional taper group (*P* < 0.05) (Fig 4).

To further explore potential variables affecting TT improvement in endurance athletes, we conducted a subgroup analysis of training intensity, volume, frequency, duration of taper, and type of taper pattern. The results showed that a tapering strategy with a reduction in training volume of 41–60% that maintained training intensity and frequency, lasted ≤ 21 days, and used progressive or step tapering could significantly improve the TT of endurance athletes (*P* < 0.05; Table 2).

1. Training intensity subgroup analysis showed that maintaining training intensity had a larger and significant overall effect on TT improvement (SMD, −0.55; 95% CI, −0.79 to −0.31; *P* < 0.05), while decreasing training intensity did not improve TT (SMD, 0.25; 95% CI, −0.67 to 1.17; *P* > 0.05) (Table 2).
2. Training volume subgroup analysis showed that a reduction in training volume of 41–60% achieved the largest and most significant improvement in TT (SMD, −0.77; 95% CI, −1.23 to −0.30; *P* < 0.05). The overall effects for the ≤ 20%, 21–40%, and ≥ 60% groups were smaller and did not reach significance levels (*P* > 0.05).
3. Training frequency subgroup analysis showed a significant improvement in TT in the subgroups in which training frequency was unchanged (SMD, −0.53; 95% CI, −0.82 to −0.25; *P* < 0.05), compared with those in which training frequency was decreased (SMD, −0.32; 95% CI, −0.76 to 0.13; *P* < 0.05).
4. Subgroup analysis of taper duration showed that the subgroup with duration of 8–14-days experienced the largest overall effect (SMD, −1.47; 95% CI, −2.75 to −0.19; *P* < 0.05), significantly better than the other subgroups. The 15–21-day group experienced the next-largest effect, and the smallest was seen in the ≥ 22-day group. All three subgroups with durations of ≤ 21 days significantly improved their TT (*P* < 0.05). Differences between subgroups were significant (*P* < 0.05).
5. Subgroup analysis of tapering type showed that the progressive-taper subgroup saw a larger and more significant improvement in TT (SMD, −0.51; 95% CI, −0.81 to −0.20; *P* < 0.05). There was also significant effect of the step taper on change in TT performance(*P* < 0.05).

**Table 2:** Subgroup analysis of the effects of tapering on TT.

Category	N	SMD (95% CI)	*P*	*I*^2^/%	*P*_inter-group_
**Training intensity**					
**Decreased**	18	0.25 (−0.67, 1.17)	0.59	45	0.10
**Unchanged**	143	−0.55 (−0.79, −0.31)	<0.001	0%	
**Training volume**					
**≤20%**	8	−0.25 (−1.27, 0.77)	0.64	*N*	0.32
**21–40%**	40	−0.45 (−0.90, −0.00)	0.05	0%	
**41–60%**	54	−0.77 (−1.23, −0.30)	0.001	22%	
**≥60%**	59	−0.21 (−0.58, 0.16)	0.26	0%	
**Training frequency**					
**Decreased**	57	−0.32 (−0.76, 0.13)	0.16	27%	0.42
**Unchanged**	104	−0.53 (−0.82, −0.25)	0.0002	0%	
**Duration**					
**≤7 days**	105	−0.36 (−0.63, −0.08)	0.01	0%	0.02
**8–14 days**	18	−1.47 (−2.75, −0.19)	0.02	63%	
**15–21 days**	28	−0.78 (−1.43, −0.14)	0.02	24%	
**≥22 days**	10	0.69 (−0.22, 1.60)	0.13	*N*	
**Type of taper**					
**Step**	63	−0.38(−0.73, −0.04)	0.03	35%	0.60
**Progressive**	98	−0.51(−0.81, −0.20)	0.001	0%	

#### Time to exhaustion

**Fig 5:**
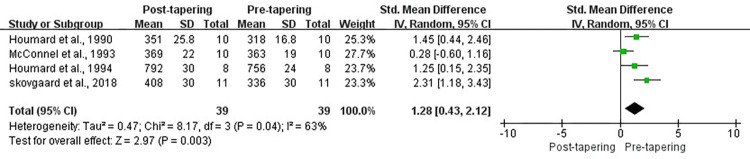
Forest plot of effect size between pre- and post-tapering for TTE.

Four studies reported data for TTE. Moderate levels of heterogeneity existed among these studies (*I*^2^ = 64%, *P* < 0.1). Results for overall effects on TTE showed a significant improvement between pre- and post-tapering (SMD, 1.28; 95% CI, 0.43 to 2.12; *P* < 0.05; Fig 5).

#### Maximal oxygen consumption

**Fig 6:**
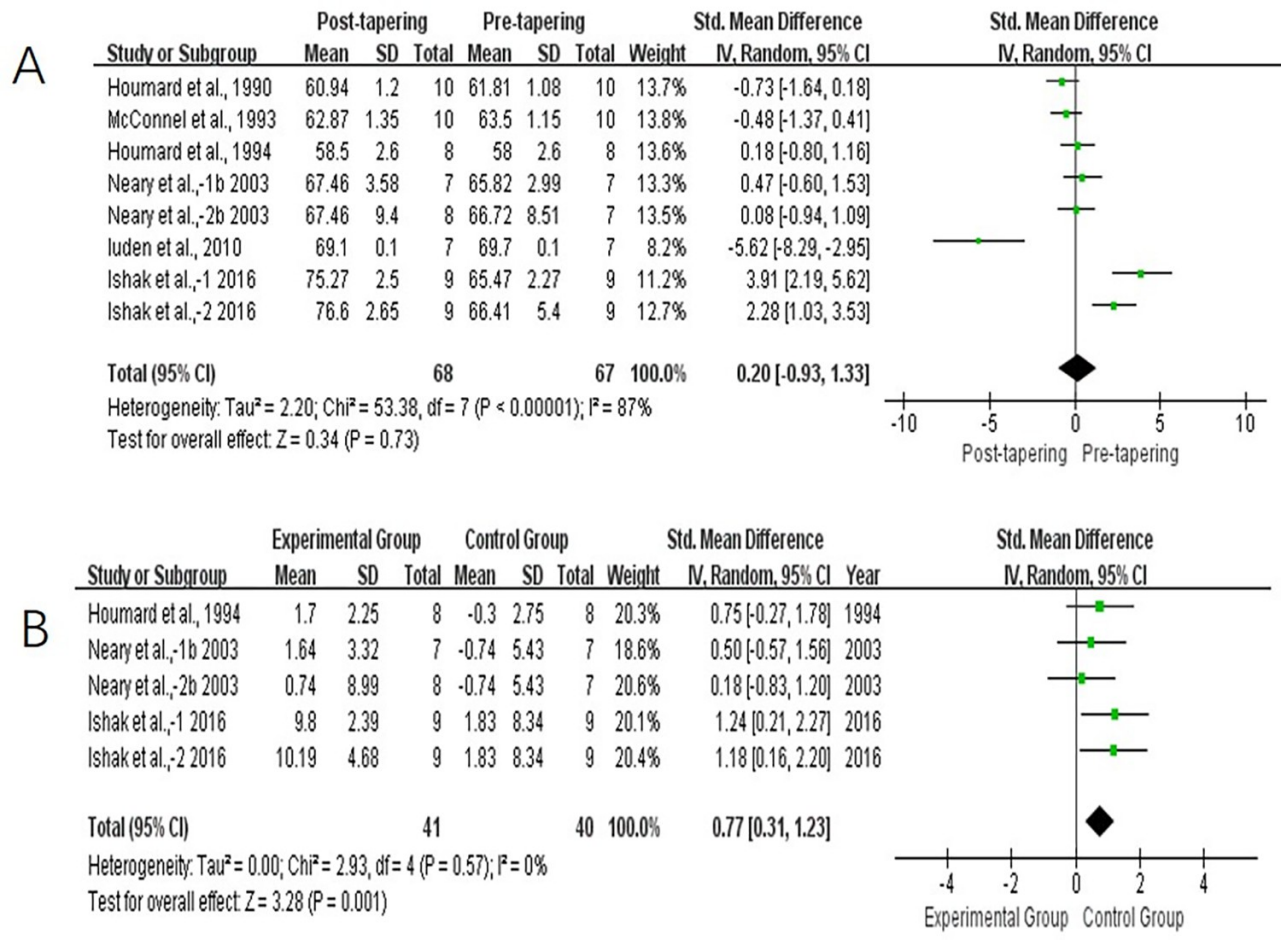
Forest plot of effect size between pre- and post-tapering for $\dot{V}O_{2\max}$ (A), and between experimental and control athletes for $\dot{V}O_{2\max}$ (B).

Six studies reported data for $\dot{V}O_{2\max}$. We observed high levels of heterogeneity among these studies (*I*^2^ = 87%, *P* < 0.1). Results showed that there were no improvement on $\dot{V}O_{2\max}$ between pre- and post-tapering (SMD, 0.20; 95% CI, −0.93 to 1.33; *P* > 0.05; Fig 6). However, the studies with both groups showed a significant improvement in the experimental groups compared with the control groups (SMD, 0.77; 95% CI, 0.31–1.23; *P* < 0.05; Fig 6), with a low level of heterogeneity (*I*^2^ = 0%, *P* > 0.1).

#### Economy of movement

**Fig 7:**
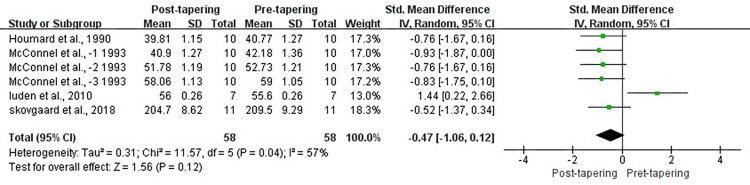
Forest plot of effect size between post and pre-tapering for EM.

Four studies reported data for EM, showing moderate levels heterogeneity among them (*I*^2^ = 57%, *P* < 0.1). Results showed that there were no improvement on EM between pre- and post-tapering (SMD, −0.47; 95% CI, −1.06 to 0.12; *P >* 0.05; Fig 7).

## Discussion

### Summary of main result

The main finding of this meta-analysis was that pre-competition tapering significantly improved TT and TTE (*P* < 0.05) and had no effects on $\dot{V}O_{2\max}$ and EM (*P* > 0.05). Further subgroup analysis results showed that pre-competition tapering combined with pre-taper overload training had a significant effect on TT improvement (*P* < 0.05), which was superior to that of conventional tapering (*P* < 0.05 for the difference between groups). A tapering strategy that reduced training volume progressively by 41–60%, maintained training intensity and frequency, lasted ≤21 days could significantly improve TT. In endurance athletes, the primary mechanism for tapering to improve endurance sports performance is to maintain or further increase aerobic exercise capacity while eliminating the psychological and physiological stress accumulated from training [23,27]. For example, cardiac pump function and work efficiency improved [10,65]; blood testosterone and blood testosterone/cortisol ratio improved significantly, whereas blood creatine kinase concentration and cortisol concentration decreased significantly [57,58,66]; heart rate to rating of perceived exertion (RPE) ratio decreased [10], and emotional state [67,68] improved. In addition, studies have shown that tapering in endurance athletes significantly increases blood volume, hemoglobin, and erythrocyte pressure volume [1], and improves mitochondrial respiratory function [68]. The improvement of these indicators enables the improvement of the oxygen-carrying and oxygen-transport capacity, maintaining a higher percentage of oxygen utilization capacity, which ultimately leads to an increase in endurance performance. The results of the present meta-analysis showed that there was no effect of pre-competition taper on $\dot{V}O_{2\max}$ (SMD, 0.20; 95% CI, −0.93 to 1.33) and EM (SMD, −0.47; 95% CI, −1.06 to 0.12) (*P* > 0.05), whereas a significant improvement was found for TTE (SMD, 1.28; 95% CI, 0.43–0.12; *P* < 0.05). The present study confirms previous findings. Busso et al. [2] indicated that long-term high-intensity sports training leads to a continuous reduction in endurance athletes’ training-induced adaptability, which is often caused by a decline in aerobic exercise fatigue tolerance caused by fatigue accumulation. TTE reflects the fatigue tolerance of these athletes [33], which is closely related to performance [37]. The increase in TTE implied that the taper played a significant role in promoting the regulation and recovery of fatigue, improving the body’s anti-fatigue ability, and delaying the occurrence of sports fatigue.

### Overload period preceding the taper

The tapering applied in conjunction with pre-taper overload training seems to be more conducive to maximize performance gains. For further exploration the training features of overload period preceding the taper, we further tracked the original research studies and found that the mean training intensity of pre-taper overload training remained at 85–95% of maximum heart rate (HR_max_), an increase of 23–26% over the mean training intensity of normal training. This was consistent with the findings of Thomas et al. [69] that an increase in pre-taper training load of approximately 20–30% was effective in improving post-taper performance. Another study found that pre-taper overload training produced higher training-induced adaptations and facilitated supercompensation [70]. The studies by Hellard et al. [71] and Le Meur et al. [72] showed that improvements in $\dot{V}O_{2\max}$ and heart pumping function of athletes who underwent overload training pre-taper were significantly higher than those of athletes who underwent conventional tapering only.

### Application strategy for taper

#### Intensity

Training intensity is an essential variable in optimising the taper. Our meta-analysis showed that a decrease in training intensity did not increase TT (SMD, 0.25; 95% CI, −0.67 to 1.17; *P >* 0.05), whereas maintenance of training intensity significantly increased TT (SMD, −0.55; 95% CI, −0.79 to −0.31; *P* < 0.05). In the included studies that examined maintenance of training intensity, the intensity of middle-distance running events (800 m, 1500 m) during tapering were maintained at 95–100% of race pace, while the endurance events such as long- and ultra-long-distance running was maintained at 85–95% $\dot{V}O_{2\max}$ (5000 m, 20 km cycling) or 82–95% HR_max_ (5000 m, 10,000 m run; 20 km cycling; half marathon) during tapering.

It should be pointed out that the above-mentioned studies mainly considered average intensity during the tapering period. The increase in TT was also closely related to the management of training intensity [29]. When the athlete recovered better from the previous overload, training could be performed at a higher intensity in the later taper phase [4]. Therefore, some studies have aeeerted that when training volume is dramatically decreased, high-intensity training (HIT) is crucial for maintaining and improving physical fitness and performance during tapering [27,73–75]. Physiological responses to this strategy may include increases in buffering capacity [56], oxidase activity, red blood cell volume, and muscle glycogen concentration [47], all of which may contribute to improved performance during a subsequent events. This is supported by training data from the most successful female cross-country skier globally. Solli et al. [64] reported that the proportion of HIT progressive increased in the the 3 weeks before the major competition, with three HIT sessions completed in the last 7 days. From the perspective of the intensity distribution characteristics included in this meta-analysis, we found that most pre-competition tapering strategies in the research used HIT. The distribution of training intensity in the included studies was characterized by alternation among HIT, moderate-intensity continuous training (MICT), and low-intensity continuous training (LICT). However, the included studies did not detail the intensity distribution characteristics. Generally speaking, as the competition day was approaching, endurance athletes tended to reduce the duration of exercise, and increase the number of exercises of a specific intensity to further increase HIT intensity to that of race pace [19], 90% $\dot{V}O_{2\max}$ [17,76], or > 90% HR_max_ [59,77]. In general, intensity distribution grew more polarized as the major competition approached [76], especially for endurance events such as long- and ultra-long-distance running. Some middle-distance events used HIT with a higher intensity than race pace (115%) for training during the tapering [29]. However, HIT at a higher intensity than race pace was less effective in improving TT than HIT at race pace [29]. For middle-distance events, race pace is already relatively high in intensity, using a continuous HIT that exceeds race pace for pre-competition training can increase the risk of injury [28]. In addition, setting HIT intensity to match race pace also helps athletes further adapt to the rhythm of the sport [28,58,71]. Therefore, in practice, especially close to the competition day, the intensity of most middle-distance endurance events tends to use race pace as HIT intensity.

#### Volume

Existing studies have confirmed that pre-competition volume reduction plays an essential role in reducing accumulated fatigue and attaining maximal performance in competition. Determining the appropriate range of volume reduction is the key factor [78,79]. The results of this meta-analysis showed that a reduction in volume of 41–60% could significantly increase TT (SMD, −0.77; 95% CI, −1.23 to −0.30; *P* < 0.05). There was a greater improvement in TT following volume reduction compared to intensity (SMD, −0.55) and frequency (SMD, −0.53). The subgroup analysis indicates that the 41–60% volume reduction subgroup prouced a large effect compared to the other three (≤ 20%, 21–40%, and ≥ 60%) subgroups. These results were consistent with those of Chen et al. [70] who adopted the gray modeling method and curve parameter estimation method to construct pre-competition load change patterns for different event groups and found that the pre-competition volume reduction for long-distance endurance events was ~46%. The above results showed that a reduction in volume of ≤ 40% was not enough to improve to TT performance, which might be due to a small volume reduction’s inability to eliminate exercise fatigue in a timely and rapid manner [80]. In comparison, a reduction in volume of ≥ 60% had no significant effect on TT improvement, which was related to the lack of quantity and quality of endurance training due to a larger volume reduction [13]. According to Jafer et al. [19], significant physiological changes (e.g., average red blood cell count, hemoglobinconcentration, and hematocrit percentages) that improve performance have been found by reducing volume up to 50% before a competition. Neary et al. [10] performed a comparative study with three reductions in training volume—30%, 50%, and 80%—in cyclists and concluded that VO_2_ and O_2_ pulse were optimized only with a 50% reduction in training volume. A meta-analysis by Bosquet et al. [27] also found that a 21–40% reduction in volume also significantly affected TT improvement (*P* < 0.05). This might be because our meta-analysis only included endurance events, whereas the meta-analysis developed by Bosquet et al. [27] included both speed events (249 athletes total) and endurance events (190 athletes total). Li et al. [79] pointed out that pre-competition volume was reduced more significantly for endurance events than for speed events. This showed that the range of volume reduction could vary by sport events. Whether the volume reduction range differed by type of endurance event (middle-distance, long-distance, ultra-long-distance) is unclear. To maximize the effect of pre-competition volume reduction on sports performance and to make the implementation of pre-competition taper strategies more pertinent and maneuverable, future research should further investigate differences in volume reduction between middle-distance endurance and long-distance/ultra-long distance endurance events.

#### Frequency

Training load can also be adjusted by changing training frequency. However, the results of studies on the effect of such change on sports performance are inconsistent, and whether a clear relationship exists between them is still unclear [79]. The pooled results of the present meta-analysis showed that a decrease in training frequency had no significant effect on TT (SMD, −0.32; 95% CI, −0.76 to 0.13; *P* > 0.05) and that maintenance of training frequency could significantly improve TT (SMD, −0.53; 95% CI, −0.83 to −0.25; *P* < 0.05). The reason might be that training frequency is often closely related to the feeling of movement and the state of competition. Maintenance of training frequency has been found to improve athletes’ pre-competition motor sensation and lead to a higher level of competitive performance, both of them would contribute to attaining optimal performance in competition [1,81]. Mujika et al. [58] compared the effects of training frequency changes on TT during 6 days of tapering in 10 well-trained male middle-distance runners; the results showed that TT decreased (1.39%) in the maintained training frequency group and a trivial (only 0.39%) in the decreased training frequency group. The investigators attributed these results to decreased motor sensation and technical proficiency due to the decrease in training frequency. That study found no difference in physiological response of athletes in different groups after tapering. In light of this, some investigators have proposed that for endurance events with lower technical content (*e*.*g*., long-distance running and cycling events), a reduction in training frequency of ~20% might be the limit to avoid a decrease in TT [82]; for endurance events with high technical requirements (*e*.*g*., swimming, cross-country skiing, and kayaking), maintenance of training frequency can effectively prevent a decline in technical efficiency [25]. It should be pointed out that the effect of decreased training frequency on performance often interacts with other training variables such as maintenance of training intensity or volume reduction; therefore, clarifying its exact impact on sports performance is difficult [27]. Although further future exploration of the interactions between changes in training frequency, intensity, and volume remains necessary, the above results showed that training frequency maintenance during tapering could effectively promote improvement in sports performance, especially in endurance events that are highly dependent on technology and high technical proficiency.

#### Duration of tapering

The key to determining optimal taper duration is a scientifically guided search for the best balance between fatigue recovery and performance improvement. The results of this meta-analysis showed that endurance athletes achieved the largest effect size during 8–14 days of taper (SMD, −1.47; 95% CI, −2.75 to −0.19; *P* < 0.05). This was similar to the meta-analysis results of Bosquet et al. [27]. The additional finding from the present study was that taper durations of ≤ 7 and 15–21 days also produced positive training effects (*P* < 0.05). The reason might be related to the different types of athletes included. According to Skorski et al. [83], in addition to $\dot{V}O_{2\max}$ and EM, anaerobic power and capacity are important factors that determine performance in road cycling. This is because in many road cycling races, first place can be decided in the final sprint, and cyclists with high anaerobic capacity and power can achieve better results [31,84]. Therefore, it may be important to determine the appropriate reduction time to optimize the power and anaerobic capacity of cyclists. Studies have indicated that 1–2 weeks of reduction is the time needed to optimize power and/or anaerobic capacity in cyclists [84,85]. When training stress decreases, there is a period of rapid adaptation and fiber hypertrophy, as evidenced by a 14.2% increase in the cross-sectional area of type IIa fiber in cyclists [86]. However, athletes who participate in cyclic sports, such as running, may require more time (2–3 weeks) to reach peak performance [27,87]. Running performance has also been found to improve after 1 or 4 weeks of tapering, but some athletes may experience negative results [27]. The increase in sports-specific muscle power during tapering tends to be greater than the improvement in aerobic fitness [88], which may be the main reason for the different durations of taper in cycling and running. The results of the present meta-analysis indicated that a period of 8–14 days appears to be the optimal taper duration for cycling and running. However, the effect of taper is also influenced by intensity, volume, frequency, and individual differences [1,89]. Recovery from a reduction in training load varies between individuals, with no clear timeline, implying that the corresponding optimal taper duration also varies [90]. Unfortunately, there are no experimental data on the difference in pre-competition training load reduction between cycling and running. Future research is needed to investigate the combination of training load reduction and its duration to find appropriate strategies for pre-competition tapering in cycling and running. Thomas and Busso [69] used a non-linear model to investigate factors influencing taper duration and found that range of load reduction and the presence or absence of overload training before tapering had a significant impact on taper duration. The results of the present meta-analysis fell within the 4–35-day taper duration range that was observed in experimental studies to effectively attain better sports performance [1]. In summary, we concluded that a taper lasting ≤ 21 days could achieve better sports performance. These data can provide a quantitative framework for training before endurance competitions. Regarding the improvement effect on performance, a taper duration of 8–14 days seems to be the best option. Still, it must be recognized that durations of ≤ 7 and 15–21 days are also effective in improving sports performance. Therefore, for specific practice, taper duration should be set according to the adjustment range of the training load and the athlete’s physiological and psychological responses.

#### Type of tapering

Taper pattern type is an organic integration of training intensity, volume, frequency, and reduction duration. Patterns are generally divided into progressive and step tapers [91]. The training load of the progressive taper presents linear or exponential changes, leading to the subdivision of this pattern into linear, fast-decay, and slow-decay tapers. The step taper is standardized to reduce training load. The results of the present meta-analysis showed that the progressive taper significantly improved TT (SMD, −0.51; 95% CI, −0.81 to −0.20; *P* < 0.05); the step taper also had an effective effect on TT (SMD, −0.38; 95% CI, −0.73 to −0.04; *P* < 0.05). This indicated that the range of reduction and speed of decrease in training load might affect improvement in TT.

Different subtypes of progressive taper have dissimilar load characteristics of changes and therefore different effects on sports performance. We did not perform subgroup analyses of the linear and exponential tapers in the present meta-analysis because an exhaustive progressive-taper scheme was not provided in the studies we included. Osman et al. [92] found that exponential taper improved TT in 1500-m athletes better than linear taper. When further comparing the effects of the fast- and slow-decay taper patterns on sports performance, most studies concluded that fast decay reduced training load more than slow decay, which was more conducive to eliminating accumulated fatigue in early training [25]. However, some studies found that compared with the fast-decay taper pattern, the slow-decay pattern could more effectively improve the performance of cyclists [69]. The reasons for these inconsistent results were not only due to the differences between athletes but also in the reduction range and speed of training load reduction caused by different taper durations. Based on the above research results, Wilson and Wilson [81] suggested that the choice of the two exponential-taper types or patterns should be combined with taper duration, with preference given to the fast-decay pattern if taper duration is short and to the slow-decay pattern in cases of longer taper duration.

### Limitations

1. The number of RCTs investigating pre-competition taper in endurance athletes was relatively small [87]. This might have led to a decrease in the quality scores of the studies included in this meta-analysis. More RCTs are needed to improve the methodological quality of the studies and assess the effect of taper moderators on field-based performance.
2. The endurance sports included in this meta-analysis were mainly cycling and running. Middle- and long-distance speed skating, cross-country skiing, and kayaking were less well represented, which could limit the application of our results to other endurance sports events. Finally, the participants of the included studies were mostly male athletes, with few female ones. The results may not be able to represent both male and female athletes in endurance events.
3. Although this meta-analysis elaborated on the effect of pre-competition taper on the performance of endurance athletes, there were specific differences in the physical and psychological responses of athletes at different levels. Owing to the limited number of included studies, we did not conduct a comparative analysis in greater depth of how tapering affected athletes of different training statuses, which might have weakened or amplified the effect of pre-competition tapering. Pre-competition tapering strategies for endurance athletes at different levels need further research and more experiments to confirm and maximize the pre-competition training effect.

## Conclusion

The tapering applied in conjunction with pre-taper overload training seems to be more conducive to maximize performance gains. Current evidence suggests that a ≤21- day taper, in which training volume is progressively reduced by 41–60% without changing training intensity or frequency, is an effective tapering strategy. Future research should further explore individualization of tapering strategy implementation and pay more attention to tapering strategies for endurance athletes of different genders, at different levels, and participating in different types of events to maximize pre-competition training effects.

## Supporting information

S1 Checklist PRISMA 2020 checklist. (DOCX)
